# Transforming care with community breast pain clinics: a validated innovative solution benefitting patients and the healthcare system

**DOI:** 10.1136/bmjoq-2025-003363

**Published:** 2025-08-20

**Authors:** John Robertson, Thilan Bartholomeuz, Veronica Rogers, Kevin Clifton, Izaak Gilchrist, Emily Griffiths, Mark Sibbering

**Affiliations:** 1 University Hospitals of Derby and Burton Foundation Trust, Derby, UK; 2 Mid-Nottinghamshire Place Based Partnership, Nottingham, UK; 3 Chesterfield Royal Hospital NHS Foundation Trust, Chesterfield, UK; 4 Edge Health, London, UK

**Keywords:** Community Health Services, Clinical Audit, Pain

## Abstract

**Rationale:**

Literature shows that breast pain alone has no significant association with breast cancer. Currently, patients experiencing these symptoms are often referred to breast cancer diagnostic clinics (BCDCs), leading to an increase in unnecessary anxiety and overutilisation of already strained secondary care resources. The East Midlands Breast Pain Pathway (EMBPP) aims to establish a new pathway that improves patient care and eases pressure on BCDCs, as well as being cost-beneficial and providing a positive patient experience.

**Aim and objectives:**

This study aims to evaluate the impact of the EMBPP on patient care, including safety, costs incurred by the health system and patient experience.

**Methods:**

The EMBPP was analysed quantitatively and qualitatively using data extracted from the community breast pain clinics (CBPCs), BCDCs, patient-reported outcome measures, clinic costs, family history data and staff interviews.

**Results:**

Breast cancer incidence within the cohort of patients with a full 12-month follow-up period was shown to be 3.7 per 1000 patients, below the population estimates in the literature. There was no delay to care for those who were diagnosed with breast cancer after attending a CBPC. The clinics were found to be cost-beneficial, with a cost-benefit ratio of 1.26 in year 1, 1.40 in year 2 and 1.56 in year 3. The pathway was positively received by patients, with 98.7% indicating that they would recommend the service.

**Conclusion:**

Following on from previous audits and analysis of the EMBPP pathway, this national evaluation has shown that CBPCs are effective across multiple Cancer Alliances, National Health Service (NHS) Trusts and demographics. The CBPC offers a positive patient experience and is cost-beneficial and safe, with no evidence of a delay to care for the patients.

**WHAT IS ALREADY KNOWN ON THIS TOPIC**

- Women with breast pain alone were being referred to cancer diagnostic clinics with a symptom which has no association with breast cancer. This caused unnecessary anxiety for these patients and filled one in every five cancer diagnostic appointments.

**WHAT THIS STUDY ADDS**

- The evaluation confirmed these patients have a low risk of breast cancer, and for the small number of patients diagnosed with breast cancer within 12 months of the community breast pain clinic (CBPC) appointment, there was no delay in diagnosis.

**HOW THIS STUDY MIGHT AFFECT RESEARCH, PRACTICE OR POLICY**

- The patients with breast pain confirmed, via the anonymised patient-reported outcome measures, that they are very happy with the new CBPCs, while patients with ‘red flag’ symptoms will have reduced waiting time to be seen and diagnosed in the breast cancer diagnostic clinics. Not only do the CBPCs bring cost efficiencies, but they also identify patients at increased familial risk of breast cancer, which is a frequent unmet need in primary care.

## Introduction

 Managing breast pain in primary care can be challenging and could prompt urgent referrals to breast cancer diagnostic clinics (BCDCs). Up to 70% of adult women experience breast pain, with 10%–20% reporting severe symptoms.^1 2^ NHS England (NHSE) guidance advises that breast pain alone is not indicative of cancer and does not warrant urgent referral on a cancer pathway.^3 4^ This aligns with evidence showing no association between ‘breast pain only’ and breast cancer (BC).^5^,^16^

The British Society of Breast Radiology supports NHSE’s position and states that ‘patients presenting with breast pain (general or focal) ONLY should not be offered imaging’.^17^ Despite this, patients with breast pain only are still frequently referred onto breast cancer (BC) diagnostic pathways,^2^ which may result in unnecessary anxiety and investigations.

Pathway optimisation is increasingly critical due to rising demand and the introduction of cancer care metrics, such as the faster diagnosis standard, which monitors 28-day diagnosis, 62-day referral to treatment and 31-day decision to treatment standards.^18^ NHSE Faster Diagnostic Pathway guidance^4^ advocates developing new pathways for managing breast pain to reduce diagnostic clinic congestion.

Patients with a family history (FH) of BC have been identified within the cohort of patients referred to BCDCs with breast pain only. Those with an FH of BC often perceive higher risk, though objective assessments frequently show no significant risk increase.^19^ A cross-sectional study found that only a minority of primary care physicians discuss FH BC risk (35.1%) or cancer prevention strategies (26.4%).^20^

The community breast pain clinic (CBPC) model, based on the East Midlands Breast Pain Pathway (EMBPP), aims to improve the management of patients experiencing breast pain only. It was designed to focus on patients within primary care, but at the same time to have positive impacts within secondary care for patients with ‘red flag’ symptoms. It has four key components:

1. Patients with breast pain only (excluding those with additional symptoms, a previous history of BC or mammary implants, and males)
2. Community-based clinics
3. Examined by an experienced clinician
4. National Institute for Health and Care Excellence (NICE) CG164 FH Risk Assessment

CBPCs were initially piloted in a Mid-Nottinghamshire NHS Trust, within Derby and Derbyshire Integrated Care System (ICS), followed by an expansion across the East Midlands Cancer Alliance (EMCA). This EMBPP model has been endorsed by both the NHSE Getting It Right First Time programme and the NHSE Cancer Programme and supported by the Association of Breast Surgery.

This ‘national’ evaluation was conducted to test whether implementation of the EMBPP could be scaled up and provide similar results to those seen in the previous pilots. Core questions were:

1. Does the EMBPP provide improved use of healthcare resources?
2. Is the EMBPP a ‘lower-risk pathway’? (ie, is there a low incidence of BC in the CBPC population?)
3. Is the EMBPP reproducible, transferrable and scalable across multiple Cancer Alliances (CAs), geographies and demographics?
4. Is the EMBPP accepted by patients?
5. Is the EMBPP a safe and timed pathway? (ie, is there any unnecessary delay to patients and their diagnosis?)

## Methods

### CBPC/EMBPP design

CBPCs offer primary care/community specialist clinics aiming to enhance patient care and experience while supporting system-wide improvements. The audited clinics, at varying stages of maturity, were divided into cohorts A and B. Cohort A tracked immediate clinic outcomes, follow-up data, rereferral rates to BCDCs within 3 months and cancer incidence within 12 months. Cohort B recorded immediate outcomes only.

All CBPCs adhered to the EMBPP model, incorporating its four key components and exclusion criteria.

Familial BC risk assessment was carried out according to NICE guidelines (CG164) for familial BC in primary care^21^ using the Family History Risk Assessment Software (FaHRAS). This is a risk assessment and patient management tool^22^ allowing data storage behind the NHS firewall and used information collected from a validated primary care FH questionnaire.^23^

Management recommendations for the CBPC are:

- Population risk—patient has no relatives with BC and requires no follow-up.
- ‘Near Population’—manage in primary care—patient has relatives with BC but requires no follow-up.
- Above near population—discuss with/refer to secondary care or tertiary care—patient has relatives with cancer and/or genetic mutations and requires discussion or referral to secondary or tertiary care.

There was prospective collection of a defined dataset including (i) core demographic data, (ii) CBPC outcome data, (iii) FH assessment data, (iv) patient-reported outcome measures (PROMs), which patients were invited to submit by anonymised questionnaires and (v) secondary care follow-up data relating to subsequent BCDC attendance or cancer diagnosis. Staff interviews took place in Spring 2024 to provide a qualitative assessment.

Further information is available on the CBPCs (online supplemental table 1), EMBPP model (online supplemental figure 1) and FaHRAS (online supplemental protocol 1).

### Information governance and data sharing

Each clinic registered the audit locally, carried out a Data Processing Protection Impact Assessment and signed a data sharing agreement with the evaluation partner, Edge Health. It was determined that NHS Trusts were the controllers who did the Data Protection Impact Assessments (DPIA) and signed the Data Sharing Agreement (DSA). The project used pseudonymised patient-level data as per the UK General Data Protection Regulation and in line with the Information Commissioner’s Office. Each Trust provided the defined dataset. The national opt-out was applied to the pseudonymised data shared, except for the fully anonymised PROMs data. The CBPC data, secondary care data and FH data were linked together by a common pseudonymisation key.

All data from patients seen in cohort A clinics between the respective clinics opening (earliest June 2021) and 29 February 2024 have been included. Follow-up secondary care data were collected until 30 April 2024. Data from Northern Lincolnshire and Goole NHS Foundation Trust (NLAG) only runs until 31 October 2023, with all available follow-up data updated on 31 January 2024. For cohort B, all CBPC outcome data were collected until June 2024.

### Audit and analysis

#### Edge Health

Edge Health is an independent healthcare data analytics firm based in the UK. It provides analytics, engineering and evaluation services to clients, including the NHS, and has worked with the EMBPP since 2022.

#### Audit framework

The audit was overseen by both a steering committee and operational committee (online supplemental table 2a,b, respectively).

The evaluation was designed using the NHS Triple Aim framework, which focuses the analysis on three aims: quality of care, cost benefits and patient experience.

#### Analysis

Quantitative analysis took place on the statistical software R Studio. Data received from Trusts were loaded, cleaned and linked before being analysed.

Staff interviews took place on Microsoft Teams. The interview guide used for these interviews is shown in online supplemental document 1. This was provided alongside a data pack that was produced for each centre using the data described above. The interviews were used to inform qualitative aspects of the audit, such as staff experience, and were not a critical analysis of the data collection. Staff interviews were the only part of the data collection of this audit to have been done retrospectively rather than collected during the audit period.

#### Cost-benefit analysis assumptions

Cost estimates were used in this analysis and represent annual costs for all clinics in cohort A and were based on Trust submissions. Assumptions used to inform the cost-benefit analysis included:

- That all attendances at a CBPC would have otherwise been referred to a BCDC. Some patients were still later rereferred to a BCDC, which is included in the analyses.
- Costs are to be the same throughout the audit for each site, except for removing fixed costs after year 1.
- To provide annual values, the costs at each site have been scaled to the number of months a clinic was open in a 12-month period.
- For diagnostic imaging, assumptions were that 0.64 mammograms are completed per BCDC referral (£66.49 reference cost) and 0.27 ultrasound scans per BCDC attendance (£67.20 reference cost).^24^

## Results

### Patient population

7326 attendances were logged across nine CAs and 17 centres (online supplemental table 1). Patients had an average age of 48 years (range 16–92 years) and an average Index of Multiple Deprivation (IMD) score of 6 (range 1–10), where 1 represents the most deprived areas.

Regarding ethnicity, 54.6% of patients were white, 5.5% Asian, 3.0% mixed, 1.6% Black and 1.6% ‘Other’ (ie, their ethnicity did not fit the defined categories). Due to data collection issues, 33.7% of patients’ ethnicities are unknown. Among patients with known ethnicity, 17.7% identified as non-white, while 82.3% identified as white.

Further details of patients’ age, IMD and ethnicity are in online supplemental table 3.

### Patient outcome

**Figure 1:**
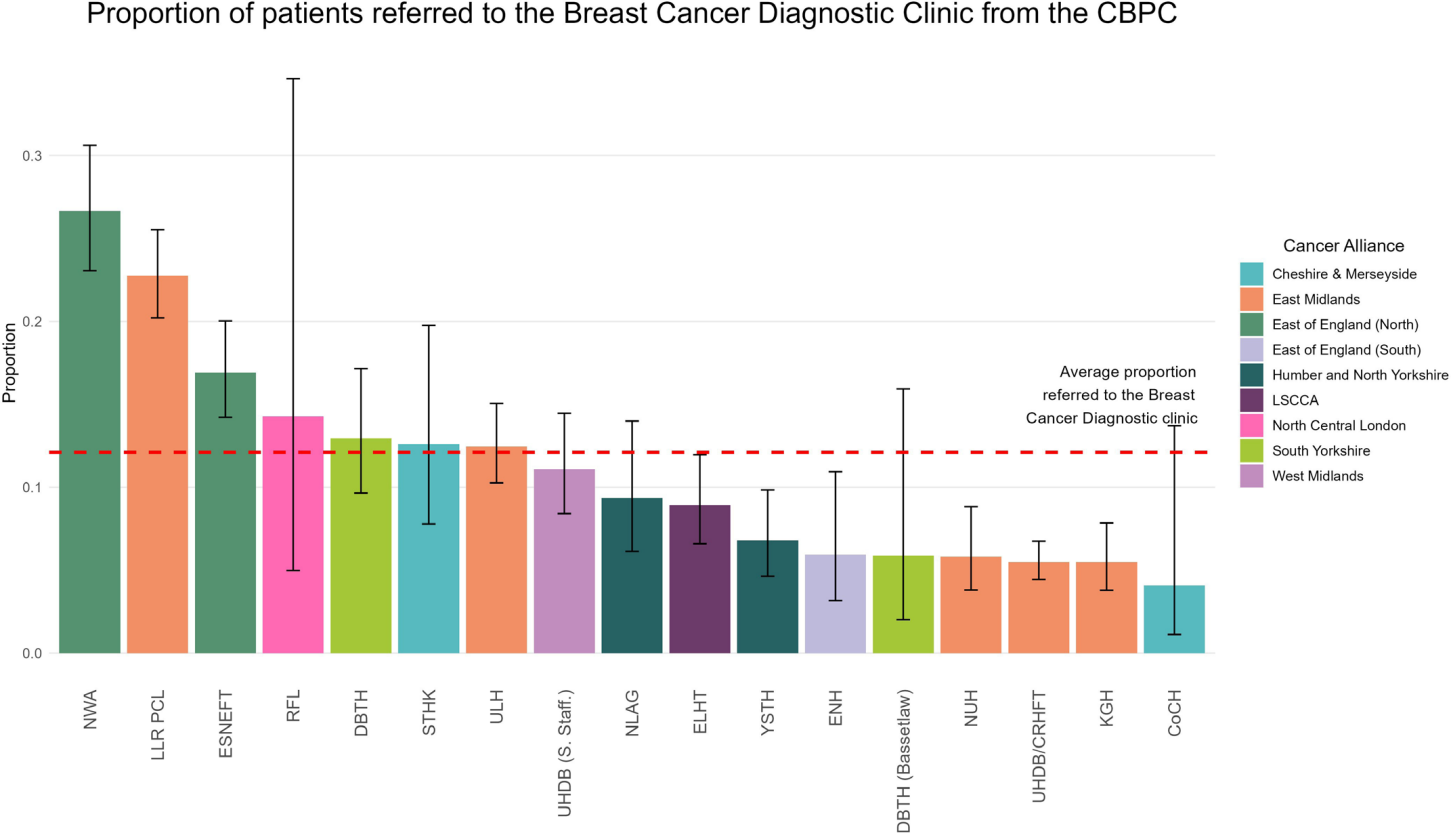
Proportion of patients referred to the BCDC from the CBPC, by Trust. BCDC, breast cancer diagnostic clinic; CBPC, community breast pain clinic; LSCCA, Lancashire and South Cumbria Cancer Alliance.

For all 7326 patients, 6439 (87.9%) were discharged following their CBPC appointment without needing further referral, while 887 (12.1%) required onward referral to the BCDC. Figure 1 shows the variability in referral rates between sites.

Cohort A patients (n=7205) had a minimum of 3 months follow-up data. BCDC appointments can result from three sources: direct CBPC referral, GP rereferral and following breast screening. Across cohort A centres, 872 BCDC appointments took place. 824/872 (94.5%) of these appointments took place after direct referral from the CBPC; 41/872 patients were subsequently rereferred by their GP: this represented 41/7205 (0.6%) of the total CBPC patients being rereferred by their GP to a BCDC within 3 months. 7/872 patients (0.8%) were referred via screening.

#### FH risk assessment

**Figure 2:**
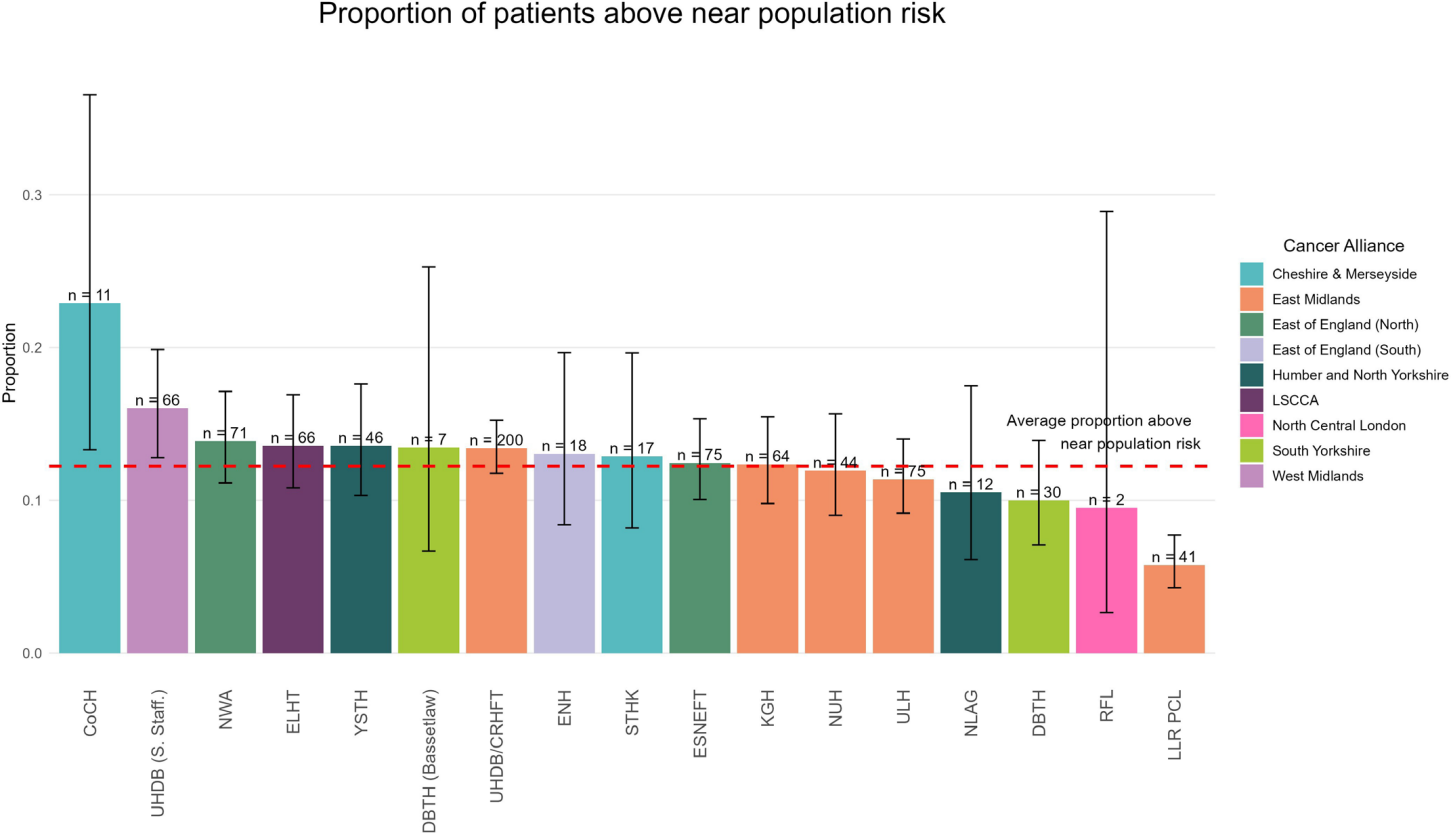
Proportion of patients requiring further management, by Trust.

Of 6903 (94.2%) patients who received a risk assessment, 68.4% (4720) had no relative with BC (population risk). 19.4% (1338) had at least one relative with BC, but that did not significantly raise their BC risk (near population risk) and required no further follow-up. 12.2% of patients (845) required further assessment due to their FH. This is shown in figure 2.

### Patient safety

It is important for patient safety that the CBPCs represent a low-risk pathway.

#### BC diagnoses

Following CBPC attendance, 24 cancers were diagnosed, including four patients who were ineligible for the clinic. A standard operating procedure (SOP) has been created to minimise the attendance of patients at higher BC risk, such as those with a personal history of BC, by specifying exclusion criteria and implementing triage measures. The SOP also outlines protocols for managing patients with a prior personal history of BC who still attend CBPCs.

#### BC incidence

The incidence of BC is 2.8 per 1000 (CI 1.8 to 4.3) for the entire eligible cohort (7324 patients, 20 cancer diagnoses). This rises to 3.7 per 1000 patients (95% CI 2.2 to 6.2) when only looking at patients who had a full 12-month follow-up period (3819 patients, 14 cancer diagnoses) (table 1).

**Table 1:** Incidence rates of breast cancer for eligible patients

	All eligible patients n=7324	Eligible patients with full 12-month follow-up n=3816
Diagnoses	20	14
Incidence per 1000	2.8	3.7
95% CI	1.8–4.3	2.2–6.2

#### Direct CBPC referrals

Of the 11 eligible patients who were diagnosed with BC following direct referral from the CBPC to the BCDC, seven patients had BC that was ipsilateral to their breast pain, two patients had contralateral BC, and for two patients the site of cancer in relation to initial breast pain was unknown.

#### Subsequent referrals through asymptomatic screening

Six eligible patients were subsequently diagnosed with BC following breast screening. All had normal clinical examinations at the CBPC, and all had been advised regarding breast screening. Two of these patients had pain, which was ipsilateral to the subsequent BC, while four had contralateral breast pain to the cancer. Five were diagnosed with invasive BC, and one with ductal carcinoma in situ.

#### Subsequent rereferrals by the GP

Three eligible patients were rereferred by their GP after attendance at the CBPC. In none of these patients was the site of the pain concordant with their breast pain. All these patients presented with new symptoms, and all were diagnosed with invasive BC.

Further details of patient eligibility and cancer diagnoses are shown in online supplemental table 4.

### Patient experience

Table 2 highlights the patient’s experience of the CBPC.

**Table 2:** Patient-reported outcome measures

Question	Answer		
	*Yes*	*No*	*Not sure*
Did you find the breast pain advice you received helpful?	*6481 (97.5%)*	*142 (2.1%)*	*25 (0.4%)*
Did you find the information regarding your personal risk of developing breast cancer helpful?	*6352 (96.6%)*	*92 (1.4%)*	*133 (2.0%)*
Did you feel reassured by the breast pain advice you received?	*6529 (98.0%)*	*77 (1.2%)*	*54 (0.8%)*
	***Extremely likely/likely***	***Not sure***	***Unlikely/extremely unlikely***
How likely are you to recommend this service to friends and family if they had troublesome breast pain?	*6465 (98.7%)*	*71 (1.1%)*	*14 (0.2%)*

**Figure 3:**
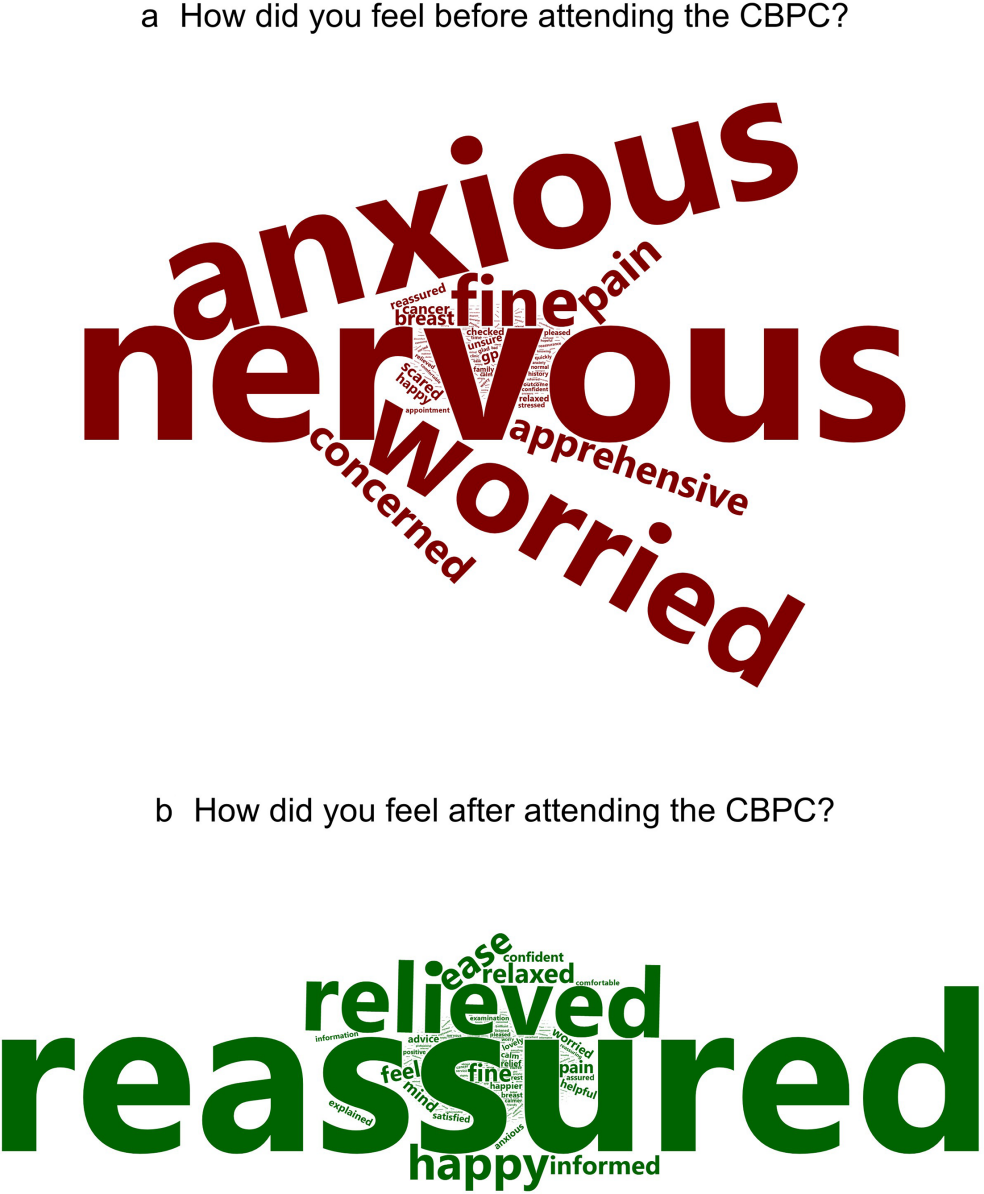
Qualitative PROMs responses. CBPCs, community breast pain clinics; PROMs, patient-reported outcome measures.

Figure 3 displays a patient’s feeling pre- and post-CBPC attendance. There is a high frequency of negative feelings prior to and positive sentiments after CBPC attendance.

 Full details of the PROMs are shown in online supplemental table 5.

### Provider economic benefits

#### Quantified benefits

In cohort A, 3385 BCDC appointments are expected to be avoided in a clinic’s first year. Therefore, using reference costs adjusted for inflation, this is a predicted saving of £809 421. For mammograms, there is a saving of £143 113, and for ultrasounds, there is a saving of £61 417. This results in a total cost-saving across all clinics of £1 013 952 in the first year of operation.

#### Cost-benefit analysis

Using the benefits quantified as part of this evaluation, the benefit to cost ratio is 1.26 in year 1. Therefore, for each £1 spent, the health system receives £1.26 in benefits. In year 2, the benefit to cost ratio rises to 1.40. In year 3, the benefit to cost ratio rises to 1.56.

CBRs for individual sites by year are shown in online supplemental table 6.

## Discussion

This report covers the expansion of the EMBPP across multiple sites, geographical localities and CAs. According to catchment population figures,^25^ there were ~3.4 million eligible female patients living in the evaluated areas (online supplemental table 7), which represent 15.3% of the population of females aged 15+ across England in 2020. The EMBPP implementation provided improved patient care and better use of healthcare resources and was accepted well by patients. The pathway caters to lower-risk patients, and there was no delay to diagnosis even with a low incidence of BC within the patient cohort.

The effectiveness of promotion of the NHS Breast Screening Programme at CBPCs has been demonstrated through six BC diagnoses via this route. This signposting increases the clinic's safety and has multiple benefits to the health system. Mammographic screening has been shown in randomised controlled trials to reduce BC mortality in women between 50 and 70 years. Clinical trials have also demonstrated the benefits of BC screening in younger women identified to be at increased risk of BC through a significant FH. The FH01 study^26^ recruited women aged 40–49 years, and the FH02 study^27^ recruited women aged 35–39 years, all with an increased risk of developing BC due to their FH, and demonstrated likely mortality reductions and earlier stage of detection benefits, respectively. Younger women identified by the EMBPP as at increased risk due to their FH may benefit from access to earlier breast screening.

Importantly, this paper reports on patient safety of the EMBPP. The BC incidence in cohort A patients should be equal to or less than the literature and expected population levels. The BC incidence rate in this study, when considering only patients who have complete 12-month follow-up, is 3.7 per 1000 (CI 2.2 to 6.2 per 1000). This is in keeping with the incidence rate reported in the literature which is 4.6 per 1000.^5^,^16^

Most BCs (11/20) detected in cohort A were diagnosed after direct referral from the CBPC. 6/20 were diagnosed through mammographic screening, and 3/20 were diagnosed through subsequent rereferral by their GP with new breast symptoms.

BC in 9/20 patients was found to be ipsilateral and concordant with their breast pain; in 9/20 it was not concordant, while the laterality in two patients was unreported. This also reflects the literature in which half the cancers diagnosed were concordant and half non-concordant, showing BC was an incidental finding.

Together with the literature, this data provide a consistent, reproducible and reassuring theme that patients presenting with breast pain only have a low incidence/risk of BC at and within 12 months of the CBPC appointment. This, along with the effectiveness of the CBPC at identifying such patients, is consistent with the previous audits conducted by the EMBPP team in Mid-Nottinghamshire,^24^ Derbyshire^28^ and EMCA.^29^

Another major finding is that across all measures of patient experience, the service was rated very highly across all centres. 98.7% of patients expressed that they would be either extremely likely or likely to recommend this service (table 2 and online supplemental table 5). Patients liked the service, and anxiety and concerns that are often associated with a BC diagnostic pathway are relieved (figure 1). This is emphasised by the very low (0.6%) subsequent referral rates to the BCDC within 3 months.

A key requirement of the EMBPP is assessing FH risk following NICE CG164 guidelines. Among CBPC attendees, 12.2% were found to have an increased BC risk. Since these data are not routinely collected in primary care, the service identifies an unmet need, enabling these patients to access interventions like breast screening, genetic testing and risk reduction strategies. Meanwhile, 87.8% of patients not at increased risk could be reassured, potentially alleviating anxiety linked to their breast pain. An important part of the risk assessment is that patients are given a copy of their risk assessment outcome.

Finally, this paper reports health economic analyses of the clinics, where the cost-benefit ratio rose from 1.26 in year 1 to 1.40 in year 2 and to 1.56 by year 3. This savings progression shows that as the efficiency and experience of sites improve with time, the benefits increase. The savings are largely driven by the reduction in BCDC referrals and a reduction in the use of diagnostic imaging. While this clearly saves money, it will also relieve pressure on the cancer diagnosis system. This aligns with the NHS Faster Diagnosis Standard guidance.^4^

The above findings on patient safety, experience and economic benefits of these clinics across multiple CAs have shown that the EMBPP is reproducible, transferrable and flexible. The flexibility and transferability are evidenced as all clinics have slightly different care models; for example, for the ‘experienced clinician’, some centres have used advanced nurse practitioners, some GPs with a special interest, hospital doctors or physician associates. The choice of this experienced clinician did not appear to have a significant impact on patient experience or safety. Another stipulation of the pathway is that clinics are held in a primary care or community care setting, which contributed to some of the cost variation.

There was variation between centres both in the proportion of referrals made to the BCDC and staffing costs, leading to some centres having lower cost-benefit ratios. The data does not show a conclusive association between staff groups and referral rates. One hypothesis is that the referral rates may reflect the seniority and number of years of clinical experience in managing breast pathology. They may also relate to the effectiveness of referral mechanisms and triage processes at different sites to ensure that patients with breast pain are only seen at CBPCs.

This audit has numerous strengths. The pathway was prospectively defined, followed the same protocol as the Derbyshire^28^ and East Midlands pilot^29^ and collected the same prospectively defined dataset. An experienced external, independent evaluator analysed the data, which included the analysis of qualitative and quantitative data. The EMBPP has been shown to provide consistent and reproducible results across multiple CAs, sites and staffing models.

There are also some limitations to the findings of this audit. First, at the time point of this audit, we did not have a full 12-month follow-up for all patients. However, we have mitigated this by reporting those patients who did have a full 12-month follow-up, and this has shown the incidence of BC in this population of women is low. Additionally, one of the key EMBPP components is the clinic being staffed by an experienced breast clinician. This was selected to enable individual centres to implement the EMBPP in a way that fits local needs and staffing patterns. Although the model of care is reproducible, this introduces some variation in delivery across sites, such as onward referral rates to the BCDC.

Assumptions were made for the health economic analysis, and 2020/21 reference costs were inflated to 2023/24 values. Although this is a very close approximation for ‘true’ costs, there may be slight variations by Trusts, which are not reflected here. The analysis also considers the costs and benefits of the clinics over 12-month periods. However, not all clinics have been open for the full 12-month period being analysed in years 1, 2 and 3; therefore, the scaled activity data could potentially have skewed the analysis slightly.

In summary, the CBPC/EMBPP provides improved patient care, a better use of healthcare resources and has excellent PROMs. Patients with breast pain alone had a low incidence of BC, and there is no delay to diagnosis. The pathway is reproducible across multiple organisations and patient demographics, making it suitable for widespread implementation.

## Supplementary material

10.1136/bmjoq-2025-003363 online supplemental file 1

10.1136/bmjoq-2025-003363 online supplemental file 2

10.1136/bmjoq-2025-003363 online supplemental file 3

10.1136/bmjoq-2025-003363 online supplemental file 4

10.1136/bmjoq-2025-003363 online supplemental file 5

10.1136/bmjoq-2025-003363 online supplemental file 6

10.1136/bmjoq-2025-003363 online supplemental file 7

10.1136/bmjoq-2025-003363 online supplemental file 8

10.1136/bmjoq-2025-003363 online supplemental file 9

10.1136/bmjoq-2025-003363 online supplemental file 10

## Data Availability

All data relevant to the study are included in the article or uploaded as supplementary information.
