# Supplementary material for: Transforming care with community breast pain clinics: a validated innovative solution benefitting patients and the healthcare system
Source: BMJ Open Qual. 2025 Aug 20;14(3):e003363. doi: 10.1136/bmjoq-2025-003363 (PMC12366605; doi:10.1136/bmjoq-2025-003363)
Supplement: online supplemental file 1 [file bmjoq-14-3-s001.docx]

**Supplementary Document 1: CBPC National Audit - Cohort A Interview Guide**

Initial Questions:

- Please describe the design of you CBPC (from GP referral to onward secondary care referral). Why were the decisions on design made?
- How and why were the location of the clinics chosen? *Location to include care setting (acute/community/primary care) and geographic location.* Do they work? Why?
- Why did you decide to implement a CBPC?
- **What information is provided to patients before attending the CBPC? What information is provided to patients after attending the CBPC?**

[pull up slides]

Attendance at the CBPC – Slide 2:

- **How often do you hold clinics? Why was this chosen?**
- How many clinic sessions per week (including number of slots if available) were available to book between October 30th 2022 and October 31st 2023? Were any clinic sessions cancelled?
- How have patients been contacted and reminded of their appointments? Do many patients DNA?
- **How far in advance are clinics cancelled? Why are clinics cancelled?**
- How are patients with exclusion criteria identified?

Referrals from the CBPC – Slide 3/4:

- What do you think are the main drivers of referral rate over time?

Staffing of the CBPC – Slide 4:

- **What is the staffing model at your CBPC? How was this staffing model of the CBPC decided?**
- **Have any staffing changes taken place during the operation of the CBPC? Why and what effects did this have?**
- **Have the CBPC staff undergone any training specific to their role at the CBPC? What training and when?**
- Does the CBPC model work well within the trust’s current staffing setup? Why? If not, what would you change?

Referral process – Slide 4

- Do CBPC patients take priority in the 2WW pathway? How was this established? Does it always work as designed?
- Do you sign-post patients to screening programmes, where appropriate

GP education – Slide 5

- **How were GPs educated on CBPC referral prior to the establishment of the CBPC?**
- **Do you have any learnings or ideas to promote GP education?**
- **Do you have any other ideas of how an ineligible patient could end up in a CBPC?**

Family History – Slide 6

- Are you aware of any demographic factors in your area that could influence family history rates?

Staff Satisfaction:

- Are staff happy to work at the CBPC?
- Would staff recommend working at the CBPC to other Trust staff?
- What do staff like about working at the CBPC compared to other clinics?
- What do staff dislike about working at the CBPC compared to other clinics?

Patient Satisfaction:

- Are patients happier to attend a CBPC than a traditional clinic?
- Did patients express any concern about attending a CBPC rather than a traditional clinic?
- Has the usage of community clinics made it easier for patients to attend?

Positives:

- Have you found any thing particularly helpful when implementing the CBPC?
- What are the main advantages of operating a CBPC at your trust?

Negatives:

- If you were to set up a CBPC again, what would you do differently?
- Are there any disadvantages to operating a CBPC at your trust?

COSTS:

- Do the FTEs quoted for the admin role include anything outside of the running of the clinics? If so, what activities?
- Do the FTEs quoted for the clinical role include anything outside of the running of the clinics? If so, what activities?
- Do any of the FTEs quoted include travel time to the clinic? If so, how much?
