# Supplementary figures and images for: Transforming care with community breast pain clinics: a validated innovative solution benefitting patients and the healthcare system
Source: BMJ Open Qual. 2025 Aug 20;14(3):e003363. doi: 10.1136/bmjoq-2025-003363 (PMC12366605; doi:10.1136/bmjoq-2025-003363)

**Supplementary Figure 1: EMBPP Pathway**

**
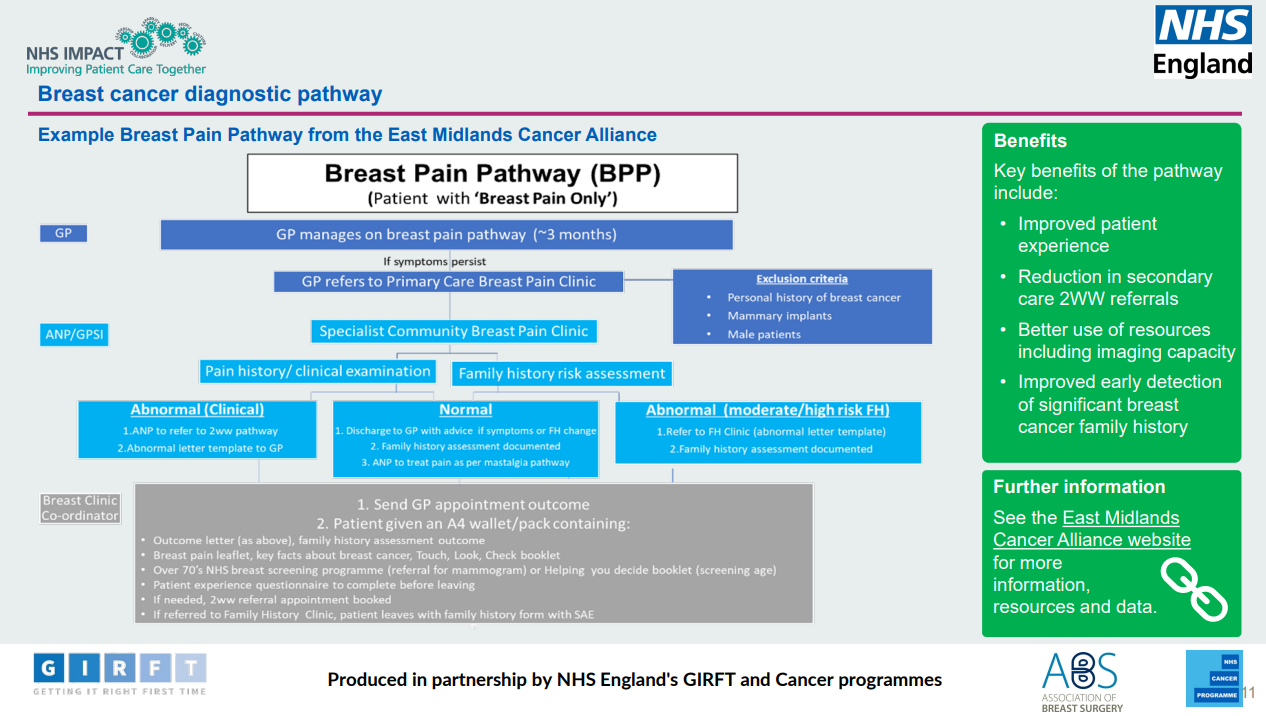
**

Supplement: online supplemental file 2 [file bmjoq-14-3-s002.docx]
