# Supplementary material for: Transforming care with community breast pain clinics: a validated innovative solution benefitting patients and the healthcare system
Source: BMJ Open Qual. 2025 Aug 20;14(3):e003363. doi: 10.1136/bmjoq-2025-003363 (PMC12366605; doi:10.1136/bmjoq-2025-003363)
Supplement: online supplemental file 3 [file bmjoq-14-3-s003.docx]

**Supplementary Protocol 1: FaHRAS protocol**

The FHQ and guidance were sent out to the patient with their appointment letter to complete and bring to their appointment. This was inputted into the FaHRAS software. There was also the option for centres to collect this information digitally using the FaHRAS-Online patient portal. The software produced a management recommendation according to the NICE Guideline CG164 (20) harmonized to the local familial cancer referral pathways as well as producing clinical letter outputs for the case notes, the referring general practitioner and the patient. The FH data, the NICE CG164 risk assessment and the patient management outputs (e.g. letters) were all stored securely behind the NHS firewall, with the service provided over the Health and Social Care Network (HSCN), and this electronic format facilitated inclusion of the FH data in the pathway evaluation.

In addition to receiving a clinical output letters for both patient and GP detailing the patient’s risk assessment, patients were also provided with a supporting information pack about breast pain, including supplementary educational information, such as breast awareness leaflets, advice on NHSBSP screening for women between 50-70 years, advice to patients over 70 years on how to access breast screening if they wished to continue to attend.
