# Supplementary material for: Transforming care with community breast pain clinics: a validated innovative solution benefitting patients and the healthcare system
Source: BMJ Open Qual. 2025 Aug 20;14(3):e003363. doi: 10.1136/bmjoq-2025-003363 (PMC12366605; doi:10.1136/bmjoq-2025-003363)
Supplement: online supplemental file 4 [file bmjoq-14-3-s004.docx]

**Supplementary Table 1: CBPCs Implementation Timeline**

| Date | Centre(s) | Acronym | Cancer Alliance | Cohort | |
| --- | --- | --- | --- | --- | --- |
| June 2021 | University Hospitals of Derby and Burton NHS Foundation Trust/ Chesterfield Royal Hospital NHS Foundation Trust (Derbyshire) | UHDB/CRHFT | East Midlands | B | |
| December 2021 | East Suffolk and North Essex Foundation Trust | ESNEFT | East of England (North) | A | |
| January 2022 | Leicester, Leicestershire and Rutland Patient Care Locally | LLR PCL | East Midlands | A | |
| March 2022 | North West Anglia NHS Foundation Trust | NWA | East of England (North) | A | |
| March 2022 | United Lincolnshire Hospitals NHS Trust | ULH | East Midlands | A | |
| June 2022 | York and Scarborough Teaching Hospitals NHS Foundation Trust | YSTH | Humber and North Yorkshire | A | |
| July 2022 | Kettering General Hospital NHS Foundation Trust | KGH | East Midlands | A | |
| July 2022 | Northern Lincolnshire and Goole Hospitals NHS Foundation Trust | NLAG | Humber and North Yorkshire | A | |
| October 2022 | Doncaster and Bassetlaw Teaching Hospitals NHS Foundation Trusts (Doncaster) | DBTH | South Yorkshire | A | |
| October 2022 | University Hospitals of Derby and Burton NHS Foundation Trust (South Staffordshire) | UHDB (S. Staffs.) | West Midlands | A | |
| November 2022 | Nottingham University Hospitals NHS Trust | NUH | East Midlands | A | |
| December 2022 | East Lancashire Hospitals NHS Trust | ELHT | Lancashire and South Cumbria | A | |
| May 2023 | East and North Hertfordshire NHS Trust | ENH | East of England (South) | A | |
| August 2023 | Mersey and West Lancashire Teaching Hospitals NHS Trust | STHK | Cheshire and Merseyside | B | |
| February 2024 | Doncaster and Bassetlaw Teaching Hospitals NHS Foundation Trusts (Bassetlaw) | DBTH (Bassetlaw) | South Yorkshire | A | |
| March 2024 | Countess of Chester Hospital NHS Foundation Trust | CoCH | Cheshire and Merseyside | B | |
| May 2024 | Royal Free London NHS Foundation Trust | RFL | North and Central London | A |  |
