# Supplementary material for: Transforming care with community breast pain clinics: a validated innovative solution benefitting patients and the healthcare system
Source: BMJ Open Qual. 2025 Aug 20;14(3):e003363. doi: 10.1136/bmjoq-2025-003363 (PMC12366605; doi:10.1136/bmjoq-2025-003363)
Supplement: online supplemental file 5 [file bmjoq-14-3-s005.docx]

**Supplementary Table 2: Names of Steering Committee and Operational Committee Members**

1. ***Steering Committee***

| Organisation | Name | Role |
| --- | --- | --- |
| EMCA | Mike Ryan | Head of Service EMCA |
| ABS | Miss Leena Chagla | President of ABS |
| ABS | Miss Sarah Downey | Vice President (President-Elect) of ABS |
| Cancer Alliances |  |  |
| EMCA | Ellie Gutteridge | Consultant Breast Surgeon, Nottingham University Hospitals NHS Trust |
| Humber & North Yorkshire | Jenny Piper | Consultant Breast Surgeon, York Teaching Hospital NHS Foundation Trust |
| West Midlands (South Staffs) | Mark Sibbering | Consultant Breast Surgeon, University Hospitals of Derby & Burton NHS Foundation Trust |
| South Yorkshire | Clare Rogers | Consultant Breast Surgeon, Doncaster & Bassetlaw Hospitals NHS Foundation Trust |
| East of England (North) | Kate Jackman | Improvement Delivery Lead, East of England Cancer Alliance |
| East of England (South) | Jane Harper | Cancer Programme Lead, Hertfordshire & West Essex Integrated Care Board (ICB) |
| Lancashire & South Cumbria CA | Inder Kumar | Consultant Breast Surgeon, East Lancashire Hospitals NHS Trust |
| Merseyside & Cheshire | Sonia Bathla | Consultant Breast Surgeon, Mersey and West Lancashire Teaching Hospitals NHS Trust |
| RM Partners (London West) | Nicky Roche | Consultant Breast Surgeon, The Royal Marsden Hospital NHS Foundation Trust |
| North Central London CA | Claire Stephens | GP & Co-Clinical Director, North Central London (NCL) Cancer Alliance |

1. ***Operational Committee***

| Organisation | Name | Role |
| --- | --- | --- |
| Association of Breast Surgery (ABS) | Carol-Ann Courtney | East Midlands Representative & member of ABS Clinical Practice & Standards Committee |
| Cancer Alliances |  |  |
| East Midlands | Julie Stone | Senior Elective Care Manager, NHS Leicester, Leicestershire & Rutland ICB |
| East Midlands | Dinesh Thekkinkattil | Consultant Breast Surgeon, United Lincolnshire Hospitals NHS Trust |
| Humber & North Yorkshire | Jennifer Smith | Consultant Breast Surgeon, Northern Lincolnshire & Goole NHS Foundation Trust & Breast Clinical Delivery Group Lead, Humber & North Yorkshire CA |
| Humber & North Yorkshire | Karen Lindley | Project Support Officer, Humber & North Yorkshire CA |
| West Midlands | Kelly Mandeley | Manager, University Hospitals of Derby & Burton NHS Foundation Trust |
| West Midlands | Lisa Rose | Advanced Nurse Practitioner, University Hospitals of Derby & Burton NHS Foundation Trust |
| South Yorkshire | Aysha Goodyear | Advanced Nurse Practitioner, Doncaster & Bassetlaw Hospitals NHS Trust |
| South Yorkshire | Claire Rogers | Consultant Breast Surgeon, Doncaster & Bassetlaw Hospitals NHS Trust |
| East of England (North) | Vanessa Hewick | Breast Services Manager and Lead Nurse, North West Anglia NHS Foundation Trust |
| East of England (North) | Habib Tafazal | Consultant Breast Surgeon, North West Anglia NHS Foundation Trust |
| East of England (South) | Fahad Matin | Programme Manager – Planned Care  East & North Hertfordshire & West Essex ICB |
| East of England (South) | Harleen Deol | Consultant Breast Surgeon, East & North Hertfordshire NHS Trust and Eastern Regional Representative ABS |
| Lancashire & South Cumbria | Suzanne Gawne | Consultant Breast Surgeon, East Lancashire Hospitals NHS Trust |
| Lancashire & South Cumbria | Tom Anderton | Senior Project Manager Lancashire & South Cumbria Cancer Alliance & NHSE Alliance Pathway Lead: Breast NHS Cancer Programme |
| Merseyside & Cheshire | Sonia Bathla | Consultant Breast Surgeon, Mersey & West Lancashire Teaching Hospitals NHS Trust |
| Merseyside & Cheshire | Ashley Breckell | Senior Quality Improvement Project Manager, Merseyside & Cheshire CA |
| North Central London | Tina Keheller | Lead Nurse for Breast Services, Royal Free London NHS Foundation Trust |
| North Central London | Muneer Ahmed | Consultant Breast Surgeon, Royal Free London NHS Foundation Trust |
| RM Partners (London West) | Lindsay Farthing | Programme Lead -Earlier & Faster Diagnosis, RM Partners |
| RM Partners (London West) | Nicky Roche | Consultant Breast Surgeon, The Royal Marsden Hospital NHS Foundation Trust |
