# Supplementary material for: Transforming care with community breast pain clinics: a validated innovative solution benefitting patients and the healthcare system
Source: BMJ Open Qual. 2025 Aug 20;14(3):e003363. doi: 10.1136/bmjoq-2025-003363 (PMC12366605; doi:10.1136/bmjoq-2025-003363)
Supplement: online supplemental file 6 [file bmjoq-14-3-s006.docx]

**Supplementary Table 3: Patient Population**

| **Descriptive statistics of patient’s characteristics** | | | | | | | | | | | | | | | |
| --- | --- | --- | --- | --- | --- | --- | --- | --- | --- | --- | --- | --- | --- | --- | --- |
| **Variable** | **N** | **DBTH**  N = 309 | **ELHT**  N = 435 | **ENH**  N = 151 | **ESNEFT**  N = 637 | **KGH**  N = 493 | **LLR PCL**  N = 949 | **NLAG**  N = 213 | **NUH**  N = 343 | **NWA**  N = 523 | **STHK**  N = 119 | **UHDB (S. Staff)**  N = 415 | **UHDB/ CRHFT**  N = 1514 | **ULH**  N = 730 | **YSTH**  N = 368 |
| **Age at attendance** | 7,199 | 46 (17, 86) | 49 (18, 88) | 48 (18, 85) | 48 (19,85) | 47 (19, 89) | 46 (16, 89) | 45 (17, 82) | 50 (17, 90) | 48 (16, 90) | 47 (16, 92) | 49 (16, 92) | 50 (16, 88) | 49 (17, 92) | 48 (17, 87) |

1. **Descriptive statistics of patient’s age, by Centre**

| **Descriptive statistics of patient’s characteristics** | | | | |
| --- | --- | --- | --- | --- |
| **Variable** | **N** | **CoCH**  N = 49 | **DBTH (Bassetlaw)**  N = 51 | **RFL**  N = 21 |
| **Age at attendance** | 121 | 52 (24, 81) | 54 (16, 79) | 49 (34, 78) |

1. **Descriptive statistics of patient’s IMD, by Centre**

| **Descriptive statistics of patient’s characteristics** | | | | | | | | | | | | | | | |
| --- | --- | --- | --- | --- | --- | --- | --- | --- | --- | --- | --- | --- | --- | --- | --- |
| **Variable** | **N** | **DBTH**  N = 309 | **ELHT**  N = 435 | **ENH**  N = 151 | **ESNEFT**  N = 637 | **KGH**  N = 493 | **LLR PCL**  N = 949 | **NLAG**  N = 213 | **NUH**  N = 343 | **NWA**  N = 523 | **STHK**  N = 119 | **UHDB (S. Staff)**  N = 415 | **UHDB/ CRHFT**  N = 1514 | **ULH**  N = 730 | **YSTH**  N = 368 |
| **IMD Score** | 5,617 | 3 (1, 10) | NA | 7 (2, 10) | 6 (1, 10) | NA | 7 (1, 10) | NA | 6 (1, 10) | 6 (1, 10) | NA | 4 (1, 10) | 6 (1, 10) | 6 (1, 10) | 7 (1, 10) |

| **Descriptive statistics of patient’s characteristics** | | | | |
| --- | --- | --- | --- | --- |
| **Variable** | **N** | **CoCH**  N = 49 | **DBTH (Bassetlaw)**  N = 51 | **RFL**  N = 21 |
| **IMD Score** | 49 | 7 (1, 10) | NA | NA |

1. **Descriptive statistics of patient ethnicity**

| ***Variable*** | | ***n*** | ***n (%)*** |
| --- | --- | --- | --- |
| ***Ethnicity*** | | ***7,326*** |  |
|  | *White* |  | *3,997 (54.6)* |
|  | *Asian* |  | *401 (5.5)* |
|  | *Mixed* |  | *223 (3.0)* |
|  | *Black* |  | *117 (1.6)* |
|  | *Other* |  | *116 (1.6)* |
|  | *Unknown* |  | *2,472 (33.7)* |
|  |  |  |  |
