# Supplementary material for: Transforming care with community breast pain clinics: a validated innovative solution benefitting patients and the healthcare system
Source: BMJ Open Qual. 2025 Aug 20;14(3):e003363. doi: 10.1136/bmjoq-2025-003363 (PMC12366605; doi:10.1136/bmjoq-2025-003363)
Supplement: online supplemental file 7 [file bmjoq-14-3-s007.docx]

**Supplementary Table 4a. Eligible and *Ineligible Cancer Diagnoses, by centre**

| **Centre** | **Number of Eligible Cancers** | **Number of *Ineligible cancers** | **Total cancers** |
| --- | --- | --- | --- |
| DBTH | 0 | 0 | 0 |
| UHDB/CRHFT | 6 | 2 | 8 |
| ELHT | 2 | 0 | 2 |
| ENH | 0 | 0 | 0 |
| ESNEFT | 2 | 1 | 3 |
| KGH | 1 | 0 | 1 |
| LLR PCL | 2 | 0 | 2 |
| ULH | 2 | 0 | 2 |
| NWA | 3 | 0 | 3 |
| NUH | 1 | 1 | 2 |
| STHK | 0 | 0 | 0 |
| UHDB (S. Staff) | 0 | 0 | 0 |
| YSTH | 1 | 0 | 1 |
| NLAG | 0 | 0 | 0 |
| **Total** | 20 | 4 | 24 |

**4b: Cancer details**

| Patient | Time between CBPC and breast cancer diagnostic clinic | Time between CBPC and diagnosis | Time between referral and diagnosis | Site of Cancer in relation to initial breast pain | Type of Cancer | Diagnosis Route |
| --- | --- | --- | --- | --- | --- | --- |
| 1* | 13 | 17 | 29 | Ipsilateral | Invasive | CBPC |
| 2 | 8 | 27 | 37 | Ipsilateral | Invasive | CBPC |
| 3 | 11 | 18 | 29 | Ipsilateral | Invasive | CBPC |
| 4 | 18 | 25 | 76 | Contralateral | Invasive | CBPC |
| 5 | 26 | 27 | 43 | Unknown | Unknown | CBPC |
| 6 | 11 | 14 | 24 | Unknown | Unknown | CBPC |
| 7 | 18 | 20 | 36 | Ipsilateral | Invasive | CBPC |
| 8 | 20 | 26 | 34 | Ipsilateral | Invasive | CBPC |
| 9 | 8 | 21 | 36 | Ipsilateral | Invasive | CBPC |
| 10 | 12 | 47 | 54 | Contralateral | Invasive | CBPC |
| 11 | 6 | 19 | 27 | Ipsilateral | Invasive | CBPC |
| 12 | 23 | 37 | 58 | Ipsilateral | Invasive | CBPC |
| 13* | 6 | 15 | 50 | Ipsilateral | Invasive | CBPC |
|  |  |  |  |  |  |  |
| 1 | 15 | 28 | 35 | Contralateral | Invasive | Screening |
| 2 | 55 | 57 | 67 | Ipsilateral | DCIS | Screening |
| 3 | 58 | 58 | 70 | Contralateral | Invasive | Screening |
| 4* | 118 | 126 | 146 | Ipsilateral | DCIS | Screening |
| 5 | 261 | 267 | 273 | Contralateral | Invasive | Screening |
| 6 | 266 | 272 | 287 | Ipsilateral | Invasive | Screening |
| 7 | 93 | 101 | 108 | Contralateral | Invasive | Screening |
|  |  |  |  |  |  |  |
| 1* | 41 | 98 | 109 | Bilateral | Invasive | GP |
| 2 | 142 | 153 | 165 | Contralateral | Invasive | GP |
| 3 | 162 | 171 | 171 | Bilateral | Invasive | GP |
| 4 | 114 | 122 | 157 | Unknown | Unknown | GP |
|  |  |  |  |  |  |  |
