# Supplementary material for: Transforming care with community breast pain clinics: a validated innovative solution benefitting patients and the healthcare system
Source: BMJ Open Qual. 2025 Aug 20;14(3):e003363. doi: 10.1136/bmjoq-2025-003363 (PMC12366605; doi:10.1136/bmjoq-2025-003363)
Supplement: online supplemental file 8 [file bmjoq-14-3-s008.docx]

**Supplementary Table 5: PROMS data**

| Response | | |
| --- | --- | --- |
| **Question** | **N** | **Statistic** |
| **Did your GP advise you that breast pain is not a symptom of breast cancer?** | 6,606 |  |
| No |  | 1,630 (25%) |
| Unsure |  | 3 (<0.1%) |
| Yes |  | 4,973 (75%) |
| **How many times have you seen your GP for this episode of breast pain prior to being referred to this clinic?** | 6,668 |  |
| 0 |  | 10 (0.1%) |
| 1 |  | 4,224 (63%) |
| 2 |  | 1,664 (25%) |
| 3 |  | 473 (7.1%) |
| 4 |  | 140 (2.1%) |
| 5 or more |  | 157 (2.4%) |
| **Patient has experienced previous episodes of breast pain that required them to see their GP** | 6,645 | 2,785 (42%) |


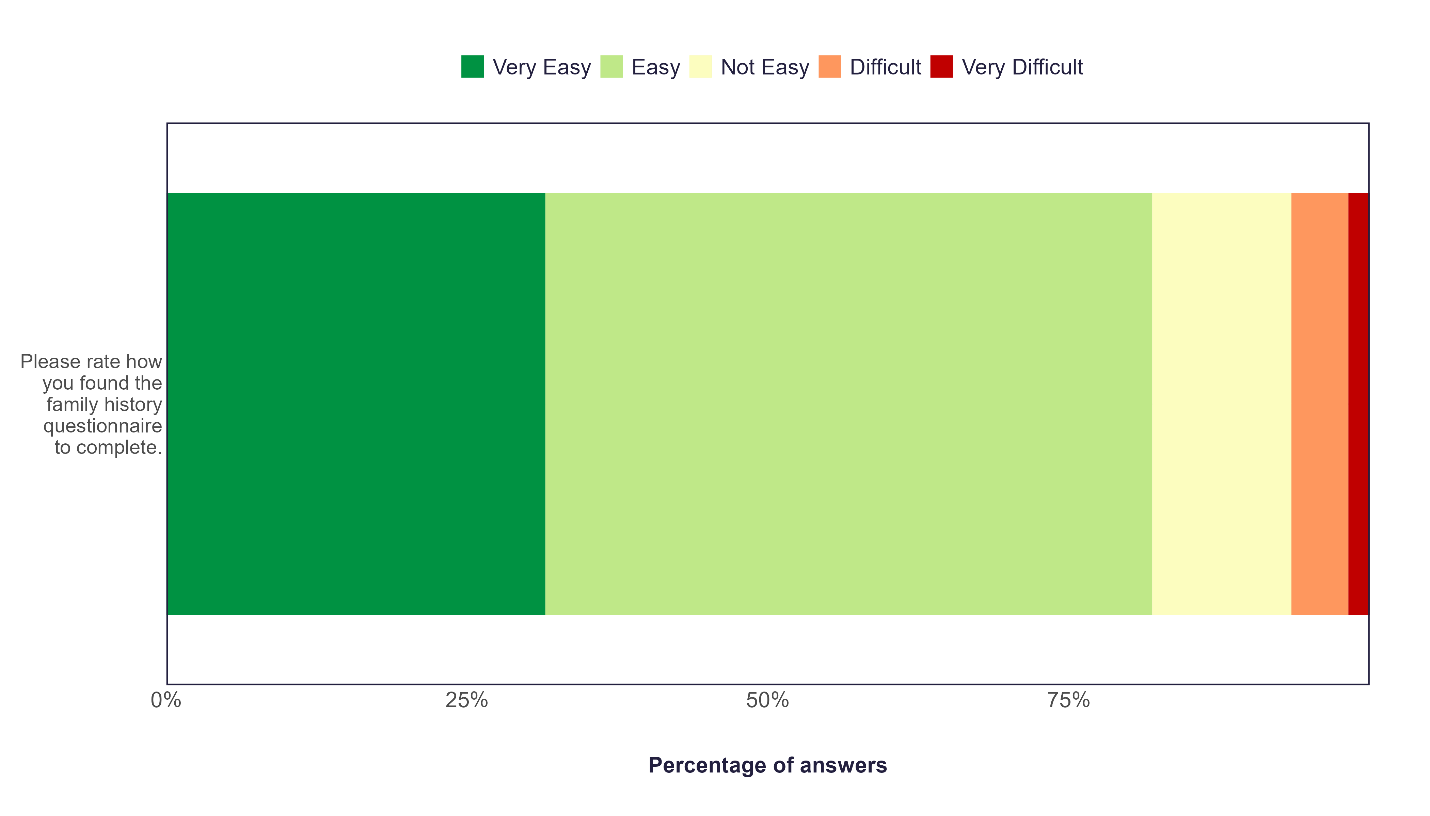


**50.4%**

**31.5%**


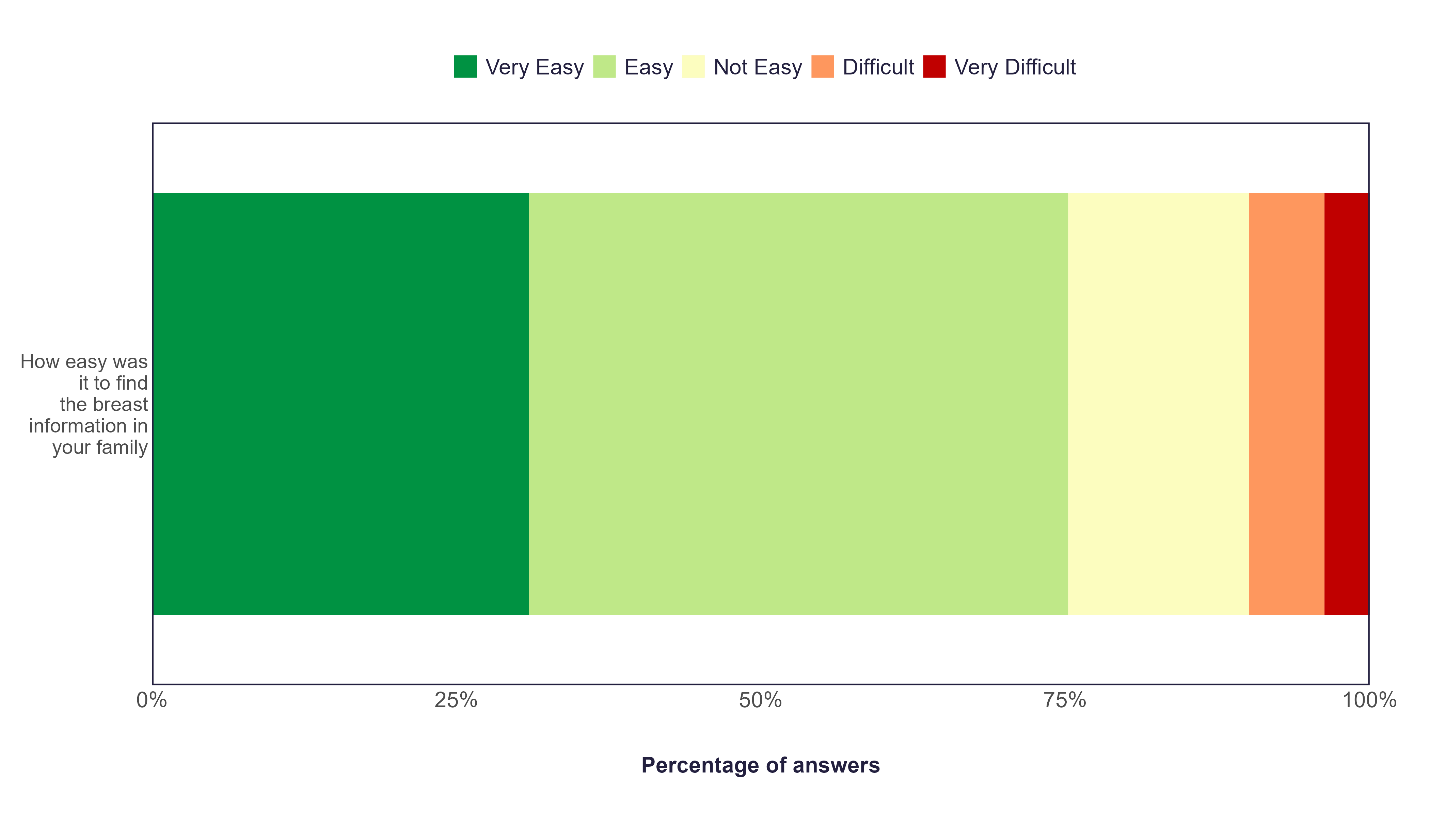


**31.0%**

**44.3%**
