# Supplementary material for: Transforming care with community breast pain clinics: a validated innovative solution benefitting patients and the healthcare system
Source: BMJ Open Qual. 2025 Aug 20;14(3):e003363. doi: 10.1136/bmjoq-2025-003363 (PMC12366605; doi:10.1136/bmjoq-2025-003363)
Supplement: online supplemental file 9 [file bmjoq-14-3-s009.docx]

**Supplementary Table 6: Cost-benefit analysis**

1. **Year 1**

| Centre | Estimated total Y1 benefits | Estimated total Y1 costs | CBR | Net benefit |
| --- | --- | --- | --- | --- |
| **DBTH** | £41,375 | £87,913 | 0.47 | -£46,538 |
| **UHDB/CRHFT** | £159,203 | £92,104 | 1.73 | £67,099 |
| **ELHT** | £97,074 | £46,138 | 2.10 | £50,936 |
| **ENH** | £41,675 | £45,884 | 0.91 | -£4,209 |
| **ESNEFT** | £65,360 | £74,447 | 0.88 | -£9,087 |
| **KGH** | £64,761 | £45,229 | 1.43 | £19,532 |
| **LLR PCL** | £105,836 | £49,824 | 2.12 | £56,012 |
| **ULH** | £86,048 | £84,638 | 1.02 | £1,410 |
| **NWA** | £69,125 | £78,593 | 0.88 | -£9,468 |
| **NUH** | £68,658 | £43,203 | 1.59 | £25,455 |
| **STHK** | £31,181 | £33,470 | 0.93 | -£2,288 |
| **UHDB (S. Staff)** | £77,053 | £29,745 | 2.59 | £47,308 |
| **YSTH** | £63,130 | £45,513 | 1.39 | £17,616 |
| **NLAG** | £43,474 | £48,819 | 0.89 | -£5,345 |
| **Total** | **£1,013,952** | **£805,520** | **1.26** | **£208,432** |

1. **Year 2**

| Centre | Estimated total Y2 benefits | Estimated total Y2 costs | CBR | Net benefit |
| --- | --- | --- | --- | --- |
| **DBTH** | £80,651 | £120,232 | 0.67 | -£39,581 |
| **UHDB/CRHFT** | £319,606 | £183,259 | 1.74 | £136,347 |
| **ELHT** | £120,460 | £53,340 | 2.26 | £67,120 |
| **ESNEFT** | £145,412 | £143,752 | 1.01 | £1,660 |
| **KGH** | £139,715 | £75,340 | 1.85 | £64,375 |
| **LLR PCL** | £211,371 | £100,098 | 2.11 | £111,273 |
| **ULH** | £195,181 | £157,808 | 1.24 | £37,373 |
| **NWA** | £115,896 | £139,367 | 0.83 | -£23,471 |
| **NUH** | £95,942 | £56,358 | 1.70 | £39,584 |
| **UHDB (S. Staffs)** | £109,733 | £40,747 | 2.69 | £68,986 |
| **YSTH** | £108,371 | £77,186 | 1.40 | £31,185 |
| **NLAG** | £58,464 | £63,855 | 0.92 | -£5,390 |
| **Total** | **£1,700,802** | **£1,211,341** | **1.40** | **£489,462** |

1. **Year 3**

| Centre | Estimated total Y3 benefits | Estimated total Y3 costs | CBR | Net benefit |
| --- | --- | --- | --- | --- |
| **UHDB/CRHFT** | £425,141 | £251,437 | 1.69 | £173,704 |
| **ESNEFT** | £160,103 | £160,651 | 1.00 | -£548 |
| **LLR PCL** | £220,666 | £105,745 | 2.09 | £114,921 |
| **Total** | **£805,910** | **£517,833** | **1.56** | **£288,077** |
