# Supplementary material for: Transforming care with community breast pain clinics: a validated innovative solution benefitting patients and the healthcare system
Source: BMJ Open Qual. 2025 Aug 20;14(3):e003363. doi: 10.1136/bmjoq-2025-003363 (PMC12366605; doi:10.1136/bmjoq-2025-003363)
Supplement: online supplemental file 10 [file bmjoq-14-3-s010.docx]

**Supplementary Table 7: Catchment Population of Trusts in the evaluation**

| NHS Trusts | Catchment Population* | Total Population |
| --- | --- | --- |
| Chesterfield Royal Hospital NHS Foundation Trust | 114,740 | 251,212 |
| Countess of Chester Hospital NHS Foundation Trust | 84,068 | 192,861 |
| Doncaster and Bassetlaw Teaching Hospitals NHS Foundation Trust | 170,048 | 391,225 |
| East and North Hertfordshire NHS Trust | 183,931 | 442,223 |
| East Lancashire Hospitals NHS Trust | 191,212 | 463,772 |
| East Suffolk and North Essex NHS Foundation Trust | 293,623 | 676,442 |
| Kettering General Hospital NHS Foundation Trust | 123,406 | 291,942 |
| Mersey and West Lancashire Teaching Hospitals NHS Trust** | 235,807 | 526,909 |
| North West Anglia NHS Foundation Trust | 217,977 | 511,469 |
| Northern Lincolnshire and Goole NHS Foundation Trust | 155,572 | 350,123 |
| Nottingham University Hospitals NHS Trust | 368,019 | 902,689 |
| Royal Free London NHS Foundation Trust | 292,947 | 684,545 |
| United Lincolnshire Hospitals NHS Trust | 220,238 | 505,116 |
| University Hospitals of Derby and Burton NHS Foundation Trust | 342,656 | 805,889 |
| University Hospitals of Leicester NHS Trust | 391,883 | 951,526 |
| York and Scarborough Teaching Hospitals NHS Foundation Trust | 222,207 | 500,191 |
| **Total** | **3,608,334** | **8,448,134** |

*Catchment defined as Females 15 years of age and above due to age group limitations

**Mersey And West Lancashire Teaching Hospitals NHS Trust is a combination of Southport And Ormskirk Hospital NHS Trust and St Helens And Knowsley Teaching Hospitals NHS Trust
